# Accelerated long-read variant calling with Clair3 for whole-genome sequencing

**DOI:** 10.1093/bioinformatics/btag181

**Published:** 2026-04-10

**Authors:** Zhenxian Zheng, Minggao He, Xian Yu, Junzhe Li, Lei Chen, Angel On Ki Wong, Jingcheng Zhang, Yekai Zhou, Ruibang Luo

**Affiliations:** School of Computing and Data Science, The University of Hong Kong, Hong Kong, China; School of Computing and Data Science, The University of Hong Kong, Hong Kong, China; School of Computing and Data Science, The University of Hong Kong, Hong Kong, China; School of Computing and Data Science, The University of Hong Kong, Hong Kong, China; School of Computing and Data Science, The University of Hong Kong, Hong Kong, China; School of Biological Sciences, The University of Hong Kong, Hong Kong, China; School of Computing and Data Science, The University of Hong Kong, Hong Kong, China; School of Computing and Data Science, The University of Hong Kong, Hong Kong, China; School of Computing and Data Science, The University of Hong Kong, Hong Kong, China

## Abstract

**Summary:**

The rapid growth of genomic data and increasing adoption of long-read sequencing technologies have rendered variant calling one of the most computationally demanding tasks in genomic analysis. Although deep learning-based methods currently outperform conventional approaches in distinguishing true variants from complex sequencing noise, they impose prohibitive computational and time requirements. To address this limitation, we present a computational framework based on Clair3 that integrates parallelized feature generation, enhanced variant phasing, in-memory read haplotagging, and GPU-accelerated neural network inference to accelerate variant calling. By dynamically optimizing the use of both GPU and CPU resources, our method achieves substantial runtime improvements without compromising accuracy. We evaluated our framework across a range of sequencing depths, diverse samples, and multiple hardware configurations. Our results demonstrate that the optimized pipeline completes variant calling for a 30× whole-genome sequence in 12–20 minutes using standard computational resources (32 CPU threads and one NVIDIA GPU), and in 12–15 minutes on an Apple Mac Studio (32 threads), which is ∼10–20-fold speedup compared with its initial release. In addition to exceptional efficiency, our method maintains state-of-the-art accuracy, achieving SNP F1-scores of 99.32% and 99.70% on 30× ONT and PacBio GIAB HG003 datasets, respectively. This work introduces a rapid, accurate, and scalable variant calling framework that effectively supports large-cohort genomic studies and time-sensitive clinical applications.

**Availability and Implementation:**

The accelerated implementation of Clair3 is open source and available at: https://github.com/HKU-BAL/Clair3/tree/gpu.

## 1 Introduction

Advances in sequencing technologies and declining costs have led to a rapid expansion in the volume of human genomic data. The identification of genomic variants represents one of the most computationally intensive steps in genomic analysis, particularly as individual sequencing data can reach hundreds of gigabytes in size (Olson et al. 2023). This scale imposes substantial demands on computational resources, both in terms of processing time and hardware requirements. The growing diversity of sequencing platforms—each producing data with distinct error profiles, read lengths, and coverage characteristics—further complicates the development of a universally efficient variant caller. In addition, large-scale initiatives such as the 1000G (Consortium 2012), gnomAD (Karczewski et al. 2020), and UK Biobank (Bycroft et al. 2018) are aggregating petabytes of genomic sequencing data, underscoring the critical need for efficient and scalable computational methods to call variants in a timely and cost-effective manner.

Long-read whole-genome sequencing (lrWGS) offers significant advantages compared to short-read technologies, particularly in resolving structural variations, characterizing repetitive genomic regions, and producing haplotype-resolved assemblies. Despite continuous improvements in raw read accuracy for both Oxford Nanopore Technologies (ONT) and PacBio platforms, the complex sequencing errors continue to challenge conventional variant calling methods for accurate small variant calling. As a result, state-of-the-art small variant callers for long-read data—such as PEPPER-Margin-DeepVariant (Shafin et al. 2021), DeepVariant (Poplin et al. 2018), Nanocaller (Ahsan et al. 2021), and Clair3 (Zheng et al. 2022)—rely on deep learning to distinguish true variants from sequencing artifacts. However, these methods are computationally demanding. For example, Clair3 incorporates ∼2 million model parameters, while DeepVariant uses an Inception-V3 network architecture with ∼20 million parameters. At typical coverages of WGS 20× to 30×, runtimes can span tens to hundreds of CPU core-hours per sample, creating practical barriers for large-cohort studies and time-sensitive clinical applications. In response, researchers are increasingly turning to accelerate data preprocessing by parallel computing and GPU (Graphics Processing Unit)-accelerated frameworks to enhance the efficiency of variant detection. Although several approaches (Gorzynski et al. 2022; Das et al. 2023, O’Connell et al. 2023, Zhu et al. 2025) have sought to reduce runtime by massively parallel processing, optimizing alignment, model inference, or post-processing, most still require substantial computational resources to achieve meaningful speedups.

To bridge this gap, we present a highly optimized, accelerated version of Clair3 that dramatically reduces runtime while maintaining state-of-the-art accuracy. Clair3 was chosen for its favorable balance of speed and accuracy, combining a lightweight pileup model for initial variant detection with a more computationally intensive full-alignment model for complex variant refinement. Our improvements include a re-implementation of parallelized genome chunking and feature generation pipeline, the integration of LongPhase (Lin et al. 2022) for rapid variant phasing, in-memory haplotagging to minimize I/O overhead, and a GPU-accelerated neural network inference module that dynamically allocates computational resources. We evaluated our framework on multiple GIAB samples sequenced with the latest ONT R10.4.1 5 kHz and PacBio Revio platforms, across a range of coverages. Our results demonstrate that the proposed workflow can process a 30× whole genome in ∼20 minutes on a Linux server with 32 CPU threads and one NVIDIA 4090 GPU, and ∼15 minutes on a macOS system with 32 threads, representing a significant speed improvement over existing methods while maintaining comparable variant calling accuracy. This combination of efficiency and precision makes our approach particularly suitable for clinical and large-scale genomic applications.

## 2 Methods

### 2.1 Overview of Clair3 accelerated variant calling workflow

**Figure 1:**
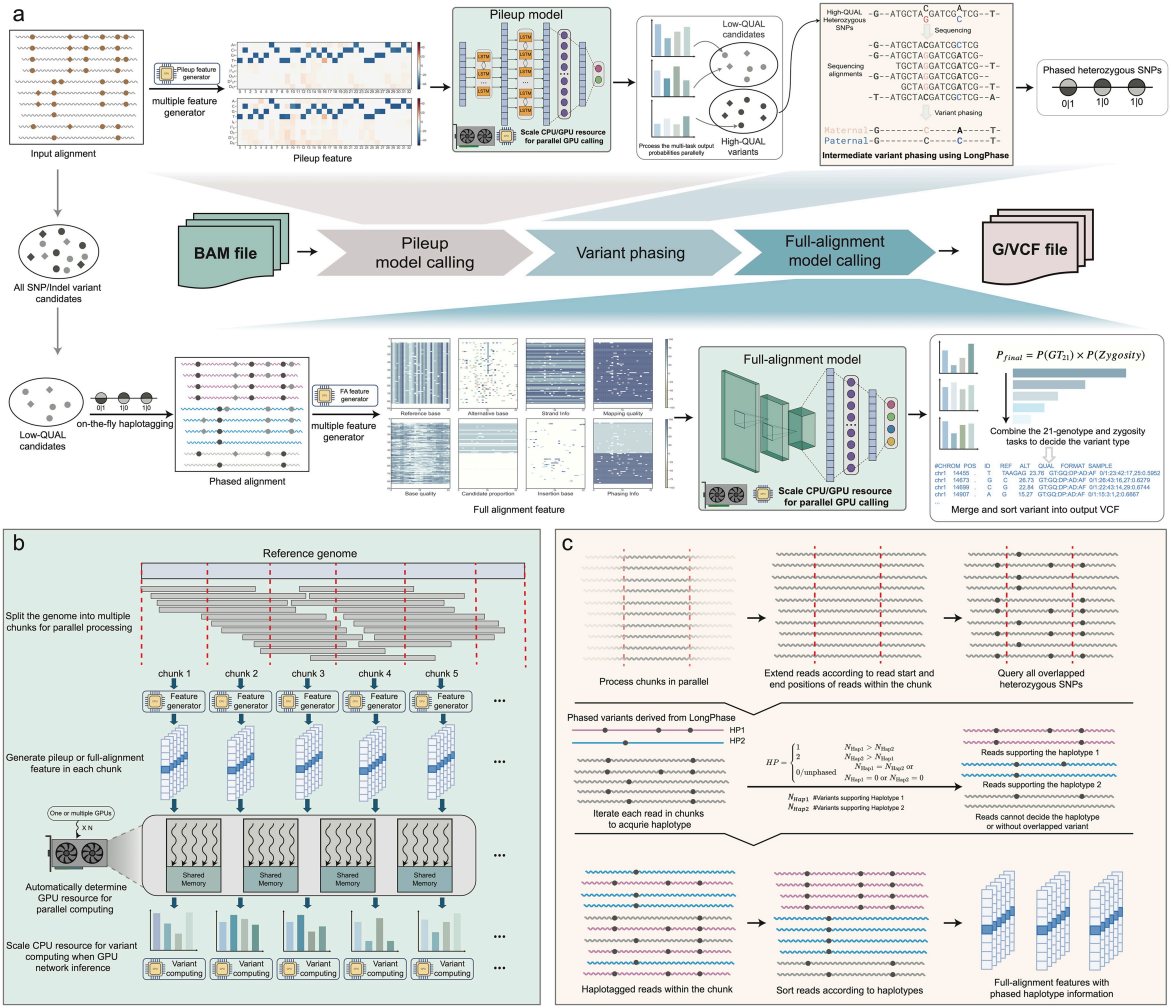
Overview of Clair3 accelerated variant calling workflow. (a) The overview of the accelerated variant calling workflow. (b) The parallel genome chunking and the parallel feature generation. (c) The workflow of our in-memory read haplotagging workflow in full-alignment calling.

As shown in Fig. 1a, Clair3 is a variant calling method designed for long-read sequencing, integrating the computational efficiency of pileup-based representation with the accuracy of full-alignment-based representation. The workflow begins by identifying variant candidates that meet minimum thresholds for read coverage and allelic fraction. These candidates undergo feature extraction, neural network-based prediction, and variant generation using probabilities derived from the network output. High-confidence heterozygous variants identified in the pileup calling step are subsequently phased using LongPhase In parallel, low-confidence pileup calls are subjected to haplotype-aware full-alignment variant calling, where reads are haplotagged to assign them to the correct haplotype. The final variant set comprising both high-quality pileup calls and full-alignment calls is exported in VCF or GVCF format. The Clair3 workflow incorporates multiple acceleration strategies, including parallel genomic chunking, streamlined variant filtering, optimized variant phasing, in-memory read haplotagging, and GPU-accelerated model inference. These enhancements collectively enable rapid variant calling in whole-genome sequencing. The following sections provide a detailed description of these acceleration techniques.

### 2.2 Genome chunking and feature generation

The genome is partitioned into contiguous 5 Mbp chunks per chromosome to facilitate parallel variant candidate discovery. Feature generation for candidate variants is confined to coordinates that lie entirely within each chunk. For read haplotagging, intervals are extended both upstream and downstream to ensure sufficient flanking sequence context is captured. Following per-chunk processing, variant calls are merged across all chunks to generate the final call set.

A critical practical consideration is the selection of chunk size. Excessively large chunks reduce the number of parallel tasks, thereby limiting concurrency and computational throughput. Conversely, overly small chunks introduce disproportionate overhead from I/O operations and task scheduling, resulting in diminishing returns. The default size of 3–10 Mbp offers a balanced trade-off and can be customized by the user. Moreover, when a region of interest is provided, or when genotyping mode is enabled, chunk boundaries are aligned to these intervals and proportionally subdivided to ensure balanced workload distribution. This approach significantly accelerates inference in scenarios involving dense targeted regions, such as amplicon sequencing.

As shown in Fig. 1b, following genome chunking, pileup features are generated in parallel for each genomic interval. To ensure feature completeness at interval boundaries, adjacent chunks are designed to overlap by a fixed number of base pairs. The features corresponding to candidate variants are subsequently fed into a neural network for prediction. This design allows computational resources to be released immediately after processing each chunk, facilitating efficient resource recycling for subsequent feature generation steps.

### 2.3 Rapid intermediate variant phasing using LongPhase

Haplotype assembly, commonly referred to as phasing, constitutes a critical step in long-read variant calling pipelines, enabling key functionalities such as variant phasing and read haplotagging. Accurate phasing is essential for distinguishing true germline variants from sequencing artifacts. Several computational methods have been developed for this purpose, including WhatsHap (Patterson et al. 2015), Margin (Ebler et al. 2019), LongPhase (Lin et al. 2022), HapCUT2 (Edge et al. 2017), etc.

WhatsHap is a widely adopted diploid phasing tool based on a fixed-parameter tractable algorithm that solves the weighted Minimum Error Correction problem. However, its performance is constrained by a practical coverage limit (typically around 15×) and high computational cost during optimal partition search. Margin employs a hidden Markov model (HMM) to integrate read and reference information for joint variant calling and phasing. While effective on PacBio HiFi data, it exhibits limited phasing continuity in repetitive or low-complexity genomic regions and remains suboptimal for noisy ONT data or highly heterogeneous samples.

In contrast, LongPhase implements an efficient two-stage graph-based phasing strategy. It first constructs phased segments locally using a directed acyclic graph of heterozygous SNPs and a greedy disjoint-path algorithm. These segments are subsequently integrated into global haplotypes by re-applying the same algorithm to a block graph connected through long-range read evidence. LongPhase demonstrates phasing accuracy comparable to WhatsHap and Margin while achieving significantly faster runtime, making it particularly suitable for large-scale and high-coverage long-read datasets. Its lightweight graph construction and path-finding algorithms reduce memory usage while maintaining high phasing continuity—even in repetitive regions, provided sufficient sequencing coverage is available.

In our implementation, we utilized the pileup-called variants and filtered for high-quality heterozygous variants, which were subsequently used as input for LongPhase to perform haplotype phasing. To enhance computational efficiency, we designed a parallelized workflow that executes LongPhase independently across different chromosomes. This approach significantly reduced runtime while maintaining phasing accuracy. Comparative benchmarking demonstrated that our pipeline achieves superior performance in both speed and phasing continuity relative to the WhatsHap phasing module.

### 2.4 In-memory read haplotagging

Read haplotagging is the process of assigning each read to its corresponding haplotype based on phased heterozygous variants. Omitting read haplotagging leads to significant performance degradation, particularly for Indel accuracy. Conventional approaches typically involve creating a temporarily haplotagged read alignment, which is then reparsered for subsequent variant prediction. However, maintaining these intermediate alignments to disk is I/O-intensive and often becomes a runtime bottleneck in long-read variant calling pipelines. Moreover, in our computational architecture, only low-confidence variant sites—comprising approximately 70% of the pileup-called variants—are subjected to haplotagging. This targeted approach significantly reduces unnecessary read-level phasing operations and minimizes computational overhead, thereby mitigating the risk of excessive haplotagging and preserving overall efficiency.

To acquire read-level haplotype information efficiently, we implemented an in-memory haplotagging strategy that operates independently of external phasing tools such as WhatsHap or LongPhase. As shown in Fig. 1c, the genome is first partitioned into chunks, which are processed in parallel on a GPU-accelerated framework. Each chunk is extended by a fixed flanking region at both ends. Within these intervals, all overlapping phased heterozygous SNPs are retrieved to construct a chunk-specific pair of reference haplotypes. Reads are extended to ensure coverage of all overlapping SNPs before haplotagging. Each read is then iteratively assigned to a haplotype based on allele consistency across overlapping variants. All overlapping heterozygous SNPs within the extended region are queried and used to construct two reference haplotypes (HP1 and HP2). For every read overlapping the phased SNP set, we compute two evidence counts. Let NHap1 denote the number of variants supporting Haplotype 1, and NHap2 denotes the number supporting Haplotype 2. The haplotype assignment for a read is determined as follows:


(1) HP={1,2,0 (unphased), if NHap1 > NHap2  if NHap1 < NHap2 otherwise

where the unphased case corresponds to $N_{\mathrm{Hap}1}=N_{\mathrm{Hap}2}$, including $N_{\mathrm{Hap}1}=N_{\mathrm{Hap}2}=0$, and 1, 2, 0 correspond to paternal, maternal, or unphased haplotypes, respectively, based on allele-specific evidence. Finally, haplotagged reads are sorted by their assigned haplotype and passed directly to a feature generation module, which produces full-alignment features in parallel for downstream variant analysis. This integrated approach significantly accelerates inference runtime and eliminates substantial disk I/O.

### 2.5 Accelerating neural network inference with GPU implementation

Clair3 GPU version is a computationally accelerated version of Clair3 that leverages GPU resources to speedup neural network inference. Unlike many existing models that rely on large and computationally intensive networks, Clair3 utilizes a compact architecture comprising approximately 2 million parameters across its pileup and full-alignment modules. This lightweight design enables efficient distribution of inference tasks across multiple GPU instances, significantly accelerating neural network prediction while maintaining high accuracy.

To maximize GPU utilization efficiency, the workflow begins with an automated estimation of available GPU resources. Computational requirements for each genomic chunk are dynamically allocated, and chunks are scheduled as independent units to minimize GPU idle time. Following feature generation, the output probabilities are batched by a dedicated subprocess. Variant calls are then generated based on the joint probabilities derived from multiple task-specific predictions, including 21-genotype classification, zygosity estimation, and Indel length inference.

### 2.6 Accelerating Clair3 variant calling in apple silicon

In contrast to traditional Linux-based computing environments, macOS systems equipped with Apple Silicon leverage a unified memory architecture that enables simultaneous data exchange between the CPU and GPU without the overhead typically associated with discrete components. This architectural advantage significantly reduces latency in memory-intensive operations, which is particularly beneficial for variant calling workflows. Due to the streamlined and hardware-agnostic workflow architecture, we were able to seamlessly port the computational pipeline from a Linux-based environment to Apple Silicon with minimal adaptation and migration. Furthermore, the portability and energy efficiency of Apple Silicon-based Mac desktops align well with the growing need for decentralized and on-site genomic analysis—enabling rapid sequencing and secondary analysis in diverse settings, from clinical point-of-care diagnostics to field research. For instance, a researcher could perform high-throughput variant calling directly in a laboratory or remote location without reliance on large computational clusters, considerably accelerating time-to-result for applications such as infectious disease surveillance or cancer genomics.

### 2.7 Benchmarking metrics

We used GIAB samples for benchmarking. For the variant calling performance, we used hap.py (Krusche et al. 2019) to compare the called variants against the true variants, and used Clair3’s ‘GetOverallMetrics’ module to generate three metrics (‘precision’, ‘recall’ and ‘F1-score’) for five categories (‘overall’, ‘SNP’, ‘Indel’, ‘Insertion’ and ‘Deletion’).

## 3 Results

### 3.1 Runtime analysis on the ONT and PacBio platforms

#### 3.1.1 ONT R10.4.1 5 kHz datasets

For the runtime analysis on the ONT sequencing platform, benchmarking experiments were conducted using the latest ONT R10.4.1 5 kHz chemistry, acquired from the ONT EPI2ME Labs using the GIAB HG003 sample. The sequencing alignments were downsampled to coverages of 30× and 50× for evaluation. We evaluated the runtime of multiple variant callers, including three releases of Clair3: Clair3 v0.1 is the initial version of Clair3 without LongPhase intermediate variant phasing and in-memory haplotagging, Clair3 v1.2 CPU mode with improved genome chunking, LongPhase intermediate variant phasing, and in-memory haplotagging, and Clair3 v1.2 GPU mode, as well as DeepVariant v1.8 in CPU and GPU modes.

**Figure 2:**
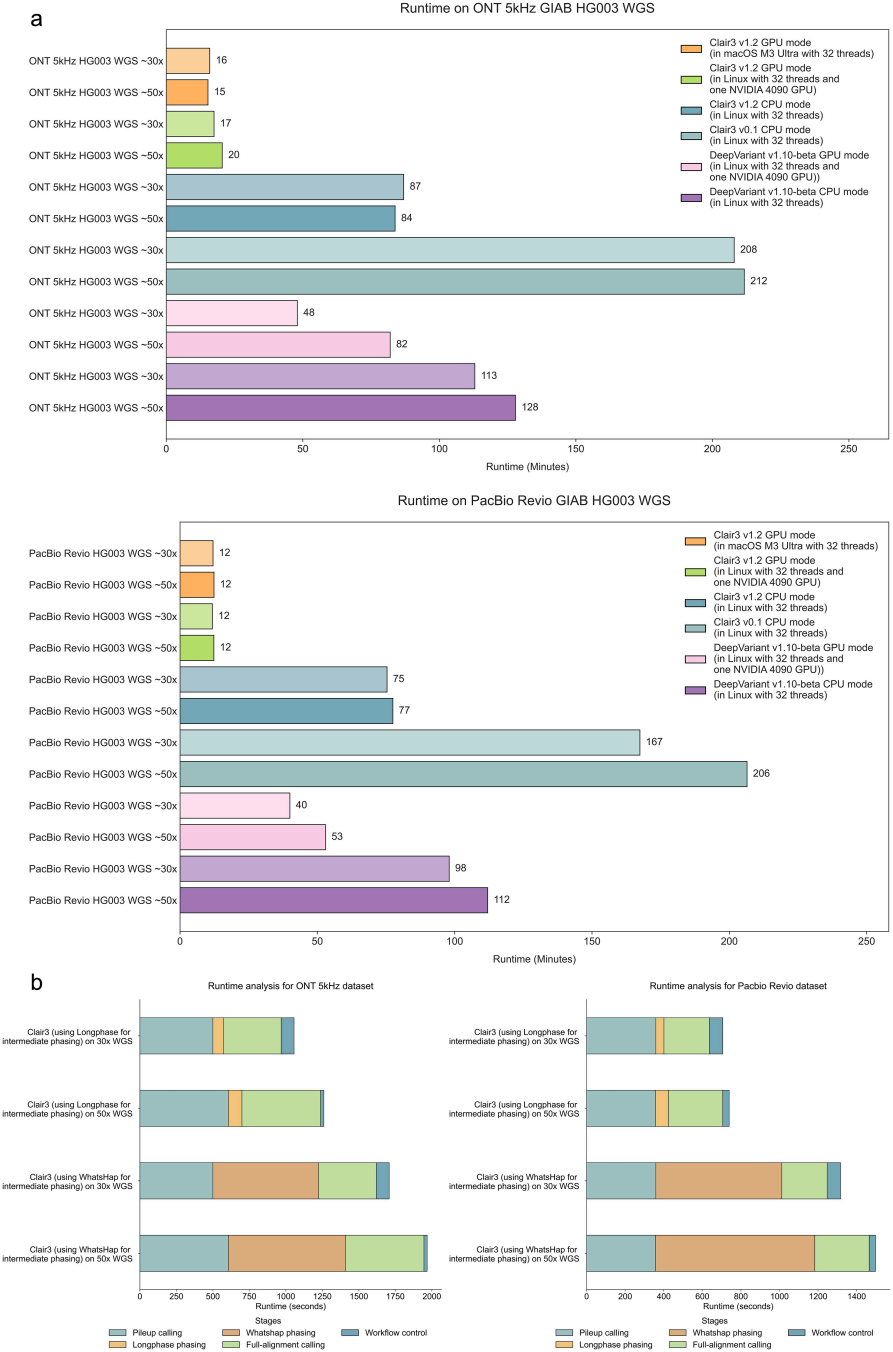
Runtime analysis in different sequence platforms. (a) Runtime comparison of different implementations of Clair3 and DeepVariant across CPU and GPU environments is illustrated. (b) The runtime analysis of Clair3 submodules is depicted.

As illustrated in Fig. 2a, under a Linux system equipped with an NVIDIA RTX 4090 GPU and 32 CPU threads, Clair3 v1.2 GPU mode consistently achieved the shortest runtime in both coverages, completing variant calling in 16 and 15 minutes in 30× and 50× whole genome sequencing, respectively. This represents a substantial improvement over Clair3 v1.2 CPU mode, which required 84–87 minutes, and the original Clair3 v0.1, which took 208–212 minutes. DeepVariant in GPU mode exhibited a runtime of 106–128 minutes, while its CPU implementation required 134–158 minutes, underscoring the runtime gap between our lightweight architecture and DeepVariant’s workflows. Notably, the GPU-enabled version of Clair3 not only outperformed all CPU-based implementations but also demonstrated superior efficiency compared to DeepVariant’s GPU mode, highlighting the effectiveness of its architectural optimizations—including in-memory haplotagging, efficient GPU resource allocation, and integration with LongPhase for rapid intermediate phasing.

#### 3.1.2 PacBio Revio datasets

We further evaluated the runtime performance of Clair3 and DeepVariant on PacBio platform sequencing data with the latest Revio sequencing system using the GIAB HG003 sample at 30× and 50× coverage. As shown in Fig. 2 and Supplementary Table 1, available as supplementary data at *Bioinformatics* online, Clair3 v1.2 GPU mode consistently demonstrated the best runtime among all callers. Under a Linux system equipped with an NVIDIA RTX 4090 GPU and 32 threads, Clair3 v1.2 GPU mode completes variant calling in 23 and 21 minutes for 30× and 50× coverage, respectively. In comparison, the CPU-only versions of Clair3 exhibited longer runtimes: v1.2 CPU required an average of 76 minutes, which is ∼3.5 times slower. And the baseline Clair3 v0.1 finished in ∼186 minutes, which is ∼10 times slower. DeepVariant v1.8 in GPU mode required 93 minutes in 30× and 118 minutes in 50×, and its CPU implementation took 128 minutes in 30× and 152 minutes in 50×, at least 4.7 times slower than GPU-accelerated Clair3. These results in ONT and PacBio affirm that GPU acceleration, combined with algorithmic refinements, significantly enhances computational efficiency in long-read variant calling.

#### 3.1.3 Runtime using the macOS machine

In runtime evaluations conducted on a macOS system equipped with an M3 Ultra processor and 32 threads, Clair3 v1.2 completed variant calling in 12 minutes for both 30× and 50× coverage data. Under equivalent CPU thread configurations, the macOS environment demonstrated consistently comparable runtime compared to the Linux machine across both sequencing platforms. On average, the macOS system achieved a 16-minute runtime for ONT data, representing a 3-minute improvement over Linux. For PacBio data, the average runtime was 12 minutes on macOS, outperforming Linux by 10 minutes. These results underscore the suitability of macOS-based portable devices for on-site and time-sensitive variant calling, enabling rapid secondary analysis in resource-limited or point-of-care settings.

#### 3.1.4 Runtime analysis of different modules

Our workflow comprises three core computational modules—pileup calling, intermediate variant phasing, and full-alignment calling—supplemented by essential workflow control steps including file I/O, heterozygous variant selection, variant sorting, and merging. To evaluate module-level efficiency, we profiled the runtime of each component within the Clair3 GPU-accelerated pipeline using both ONT 5 kHz and PacBio Revio datasets, with particular emphasis on the performance of LongPhase and WhatsHap for the phasing step.

As summarized in Figure 2b, LongPhase consistently runs faster than WhatsHap across both sequencing technologies. On the ONT 30× and 50 × 5 kHz datasets, LongPhase completed phasing in just 72/93 seconds, compared to 724/801 seconds for WhatsHap, representing a 9-fold speed improvement. Similarly, on the PacBio Revio 30× and 50× datasets, LongPhase required 42/67 seconds versus 653/826 seconds for WhatsHap, corresponding to a 13-fold reduction in runtime. Beyond phasing, the pileup calling module demonstrated high efficiency, completing in average ∼9 minutes for ONT and ∼6 minutes for PacBio to handle all variant candidates. In contrast, full-alignment calling constituted the most computationally demanding stage, requiring ∼8 minutes and ∼4 minutes for ONT and PacBio, respectively, to handle 70% pileup calls and 10% reference calls. Workflow control operations contributed minimally to runtime, with an average of ∼53 seconds, highlighting the efficacy of in-memory haplotagging and parallelized chunk processing. Collectively, these results demonstrate substantial runtime improvements over CPU-based implementations, enabling accurate and rapid long-read whole genome variant calling.

### 3.2 Performance analysis on different sequencing platforms

While computational efficiency is critical for practical usage, the accuracy of the variant calling algorithms is also important to ensure reliable variant calls. To comprehensively evaluate performance, we analyzed Clair3's variant calling performance across multiple GIAB samples using both ONT and PacBio datasets. The Clair3 ONT model was trained in HG002 using the data provided by ONT EPI2ME Labs, and the PacBio Revio model was trained in HG002 and HG004 using the data provided by the PacBio research team. All trained variants and samples are unified using Repun (Zheng et al. 2025) for enhanced model training. We compared the performance of Clair3 with DeepVariant on HG003, as both callers have excluded HG003 as a hold-out dataset for benchmarking. We also benchmarked Clair3 performance on HG001, HG002-HG004 Ashkenazim Trio, and HG005-HG007 Chinese Trio, where the truth variants have been fully validated (Wagner et al. 2022).

**Figure 3:**
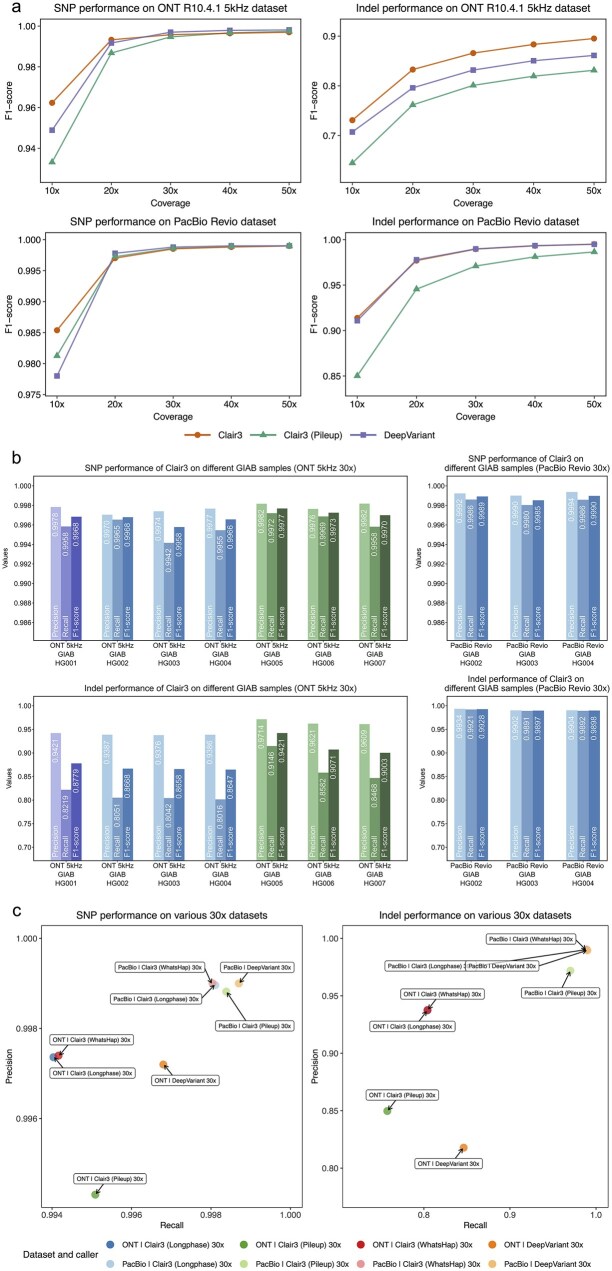
Performance analysis across different sequence platforms. (a) Performance of multiple variant callers across a range of coverages on both ONT and PacBio datasets is depicted. (b) Performance evaluation of Clair3 across multiple GIAB reference samples (HG001–HG007), each sequenced at 30× whole-genome coverage, is depicted. (c) Precision and recall of Clair3 when using LongPhase versus WhatsHap for intermediate variant phasing.

As shown in Figure 3a, Clair3 outperformed DeepVariant on the ONT platform across HG003 whole-genome sequencing data at both 10× and 20× coverages, while demonstrating comparable performance beyond 20× coverage. Specifically, Clair3 achieved 96.42%p(precision)/96.05%r(recall)/96.23%f (F1-score) and 99.46%p/99.20%r/99.33%f for SNP in 10x and 20x WGS, respectively. In comparison, DeepVariant achieved 97.34%p/92.17%r/94.68%f and 98.75%p/98.79%r/98.79%f for SNP in 10× and 20× WGS, which are 1.55% and 0.54% lower relative to Clair3 in F1-score. Similarly, for Indel detection, Clair3 also outperformed DeepVariant by 0.71% and 1.46% in F1-score at 10× and 20×, respectively. At coverages above 20×, Clair3 remained competitive with DeepVariant for SNP (average 99.64%f vs. 99.66%f) and exhibited a slight advantage in Indel calling (average 88.14%f vs. 87.11%f).

For the PacBio Revio dataset, Clair3 had competitive SNP performance and consistently outperformed DeepVariant in Indel detection across coverages from 10× to 50×. At 10× coverage, Clair3 achieved SNP 98.54%f and Indel 91.39%f, compared to SNP 97.81%f and Indel 90.74%f for DeepVariant. At 20×, Clair3 achieved 99.70%f for SNP and 97.70%f for Indel, while DeepVariant achieved 99.78%f and 97.60%f for SNP and Indel, respectively. Although DeepVariant surpassed Clair3 in SNP detection after 10×, while Clair3 outperformed DeepVariant in overall accuracy, particularly in Indel calling. Furthermore, we observed that the Clair3 pileup model achieved 93.32% and 98.13% SNP F1-scores on 10× ONT and PacBio datasets, respectively. Notably, its performance on PacBio data surpassed that of DeepVariant by 0.32%f (98.13%f vs. 97.81%f). Given that the pileup model completed variant calling in ∼6 minutes, these results highlight its strong potential for efficient and accurate analysis in low-coverage applications.

We further evaluated Clair3’s performance using ONT data from GIAB reference samples HG001 to HG007. As shown in Figure 3b and Supplementary Table 2, available as supplementary data at *Bioinformatics* online, for SNP calling, Clair3 consistently achieved high accuracy across all samples, with precision exceeding 99.70% and recall above 99.42%. Performance was peaked in HG005, which attained SNP 99.77%f. For Indel detection, Clair3 demonstrated higher precision than recall in all evaluated samples, with an average 95.02% precision and 83.61% recall across the seven samples. The relatively lower Indel recall can be attributed to challenges in complex genomic regions—such as those with low mappability, segmental duplications, or low sequence complexity—which remain difficult to resolve confidently even with long-read data. Overall, Clair3 exhibited stable and robust performance across diverse samples, underscoring the generalizability of the proposed architecture. On the PacBio platform, Clair3 was evaluated using the HG002–HG004 Ashkenazim Trio dataset. As summarized in Figure 3c and Supplementary Table 3, available as supplementary data at *Bioinformatics* online, it achieved an average 99.88%f for SNP and 99.07%f for Indel, demonstrating its high accuracy in variant detection on PacBio data.

### 3.3 Comparison of different phasing methods

**Figure 4:**
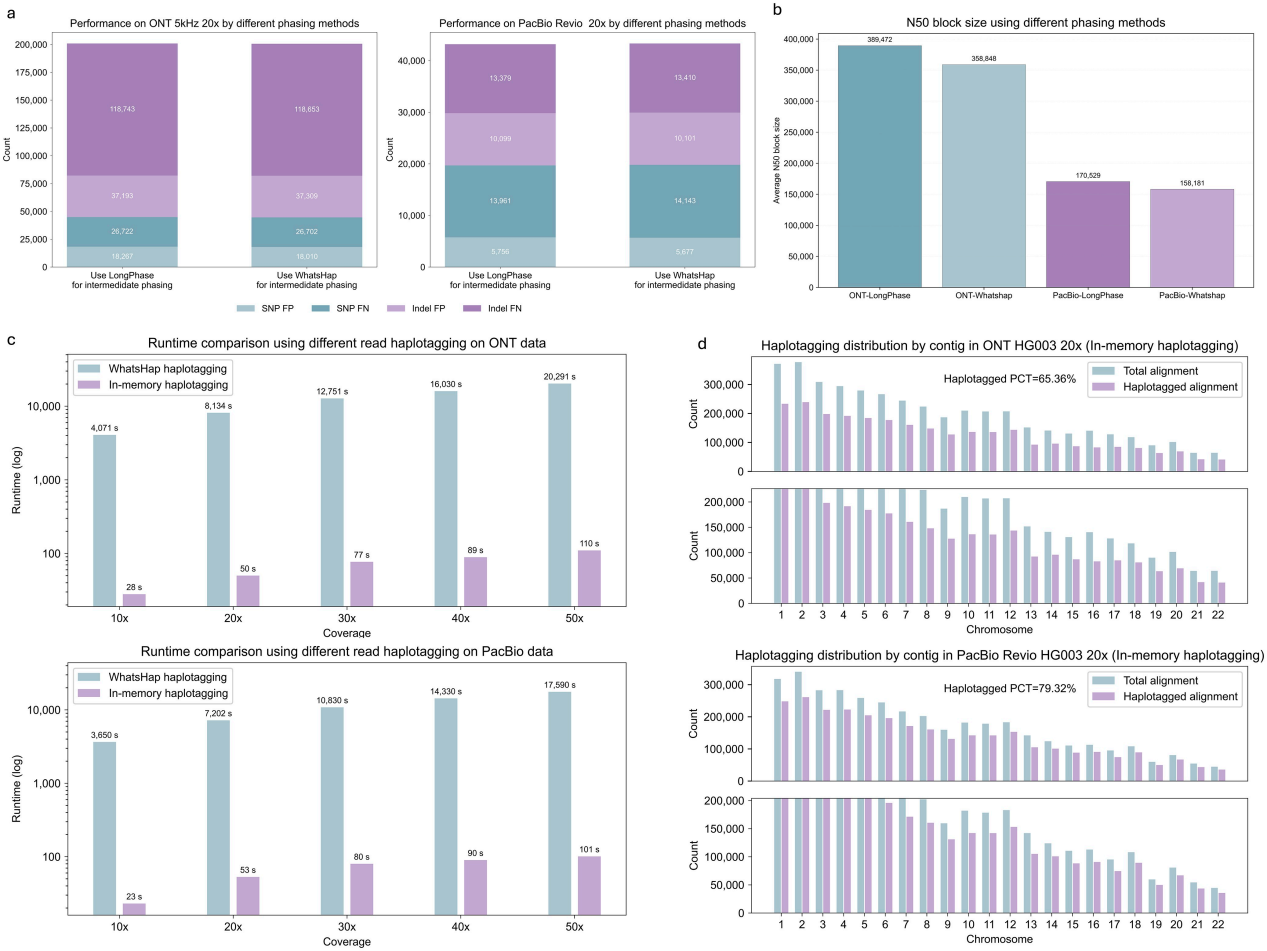
Performance analysis of different variant phasing and read haplotagging methods. (a) Performance comparison of using LongPhase and WhatsHap for intermediate phasing. (b) Comparison of phased block continuity (N50) between LongPhase and WhatsHap is depicted. (c) The runtime comparison between in-memory haplotagging versus the standard WhatsHap implementation is illustrated. (d) Read haplotagging statistics for ONT and PacBio platforms is depicted. “Haplotagged PCT” refers to the percentage of haplotagged reads within the respective subset.

As shown in Figure 4a and Supplementary Table 5, available as supplementary data at *Bioinformatics* online, we compared the performance of LongPhase and WhatsHap for intermediate phasing on both ONT and PacBio sequencing platforms. Across coverages from 10× to 50×, LongPhase achieved average 98.89%f for SNP and 84.15%f for Indel on ONT data, and 99.58% for SNP and 97.38% for Indel on PacBio data. These results are highly comparable to those of WhatsHap, which attained average 98.90%f for SNP and 84.16%f for Indel on ONT, and 99.57%f for SNP and 97.38% for Indel on PacBio. A more detailed performance comparison revealed platform-dependent differences between the two methods: LongPhase produced fewer false positives and false negatives on PacBio data, whereas WhatsHap exhibited higher accuracy on ONT data. As illustrated in Figure 3a, LongPhase demonstrated superior specificity and sensitivity on PacBio data, producing fewer false positives and false negatives (43,195) compared to WhatsHap (43,331). In contrast, on ONT data, WhatsHap exhibited a slight advantage, yielding 200,674 combined false positives and false negatives, outperforming LongPhase, which produced 200,975. Moreover, as shown in Figure 4b, LongPhase generally has better N50 phase block size compared with WhatsHap (389,472 versus 358,848 in ONT and 170,529 versus 158,181 in PacBio). Overall, while both methods achieve similar accuracy in variant phasing, LongPhase offers substantially faster integration within the variant calling workflow, making it especially suitable for time-sensitive applications. In contrast, WhatsHap retains a slight edge in haplotype-aware accuracy.

### 3.4 Performance analysis for in-memory read haplotagging

We further evaluated the read haplotagging performance of our implemented in-memory read haplotagging method with WhatsHap haplotagging submodule. Higher haplotagging percentages generally correlate with improved variant calling accuracy, as correctly assigned reads help distinguish true variants from sequencing artifacts. As depicted in Figure 4d and Supplementary Table 5, available as supplementary data at *Bioinformatics* online, our in-memory read haplotagging successfully haplotagged 65.36% of reads on the ONT 20× dataset, and 79.32% reads on the PacBio 20× dataset, outperformed WhatsHap haplotagging by 0.13% and 0.11% in ONT and PacBio, respectively. Moreover, we compared the runtime two methods. As shown in Figure 4c, WhatsHap required at least 68 minutes (average 204 minutes) to process ONT data across coverages from 10× to 50×, while our method completed the same task within 110 seconds—representing a speedup of over 100-fold. Similarly, on PacBio data, our approach performed genome-wide haplotagging in an average of 69 seconds. These dramatic efficiency gains are primarily attributable to the elimination of disk I/O bottlenecks and the implementation of optimized genomic chunking for parallel processing.

## 4 Discussion

As the cost of genome sequencing continues to decline, genomic datasets are rapidly expanding in both scale and accessibility. This growth promises to advance critical initiatives such as large-scale population genetics and personalized medicine. Long-read sequencing technologies have demonstrated advantages over short-read approaches in resolving complex genomic regions, including low-complexity sequences and low-mappability regions, that are difficult to access with short reads. These capabilities support more comprehensive variant catalogs, gapless haplotype construction, and more accurate disease association studies in previously challenging genomic contexts. However, long-read sequencing technologies, particularly the ONT platform, exhibit higher error rates compared to short reads, necessitating highly robust variant callers to ensure accurate variant detection.

This work bridges a critical gap between the superior accuracy of long-read whole-genome sequencing and the practical limitations of computational scalability. By integrating parallel feature preprocessing, efficient variant phasing, and GPU-accelerated network inference, our framework reduces the time required for highly accurate, haplotype-aware small-variant calling from hours to minutes. This efficiency enables applications at population-scale cohort analysis as well as time-sensitive clinical use. In large genomic initiatives—such as 1000G, gnomAD, the UK Biobank research program—computational efficiency is becoming as crucial as sequencing throughput. The ability to reprocess samples within minutes allows for rapid computation of datasets as sequencing chemistries, basecallers, and reference genomes continue to evolve.

The clinical implications of this work are substantial. In acute care and other time-sensitive clinical settings, turnaround time is frequently the limiting factor, while computational demands often preclude the use of sophisticated genomic analysis outside specialized centers. Our method enables accurate long-read variant calling on widely available hardware, including consumer-grade laptops and workstations, making rapid genomic analysis accessible outside high-performance computing environments. When integrated with recent advances in portable nanopore sequencing, such as rapid library preparation and real-time basecalling, our lightweight, GPU-accelerated pipeline supports an edge-computing paradigm in which high-confidence variant calls can be generated almost immediately after sequencing is complete. This capability holds promise for rapid diagnosis and timely clinical triage in resource-limited settings.

Although our approach addresses the dominant error profiles in current long-read sequencing data, performance remains challenged in technically difficult regions such as homopolymers and segmental duplications, where elevated noise levels increase false-positive candidates and compromise calling efficiency. Ongoing improvements in sequencing chemistry and basecalling algorithms are expected to improve alignment in these complex regions. Additionally, model inference constitutes the most time-consuming component of our workflow and is highly dependent on GPU computational capacity. Future efforts could explore the integration of more accurate neural architectures, such as attention-based networks, to achieve comparable accuracy with reduced model size and inference time, thereby enhancing both scalability and speed.

## Supplementary Material

btag181_Supplementary_Data

## Data Availability

The 1) links to the reference genomes, truth variants, benchmarking materials, and ONT and PacBio datasets, and 2) the benchmarked commands and parameters used in this study are available in the Supplementary Notes. All analysis output, including the VCFs and running logs, is available at http://www.bio8.cs.hku.hk/clair3/runtime_analysis_result.

## Code availability

Clair3 and its accelerated version is open source and available at https://github.com/HKU-BAL/Clair3/tree/gpu under the BSD 3-Clause license. A public Docker image hkubal/clair3-gpu: latest that can evaluate Clair3 GPU’s evaluation pipeline is also available for simplicity. Users can run the workflow seamlessly in one command with the input BAM file and reference file. Clair3 provides user interface flexibility that supports parallel computing, user-customized parameter settings, and workflow refinement.

## Computational performance

Clair3 was implemented with Python and TensorFlow and leveraged C in feature generation and in-memory haplotagging for speedup. Variant calling using Clair3 GPU version is benchmarked with other callers using the NVIDIA GeForce RTX™ 4090 GPU resources using two 12-core Intel Xeon Silver 4116 processors. The macOS setup used a Mac Studio with 32 threads for computation. The memory consumption is capped at 500 MB per process. Model training requires a high-end graphics processing unit. We used TensorFlow to train models on an NVIDIA GeForce RTX™ 3090 and 4090 GPU. We used the GNU time command to benchmark the runtime and memory usage.
