# Supplementary material for: Accelerated long-read variant calling with Clair3 for whole-genome sequencing
Source: Bioinformatics. 2026 Apr 10;42(5):btag181. doi: 10.1093/bioinformatics/btag181 (PMC13141153; doi:10.1093/bioinformatics/btag181)
Supplement: btag181_Supplementary_Data [file btag181_supplementary_data.pdf]

Supplementary Notes

Supplementary Figures

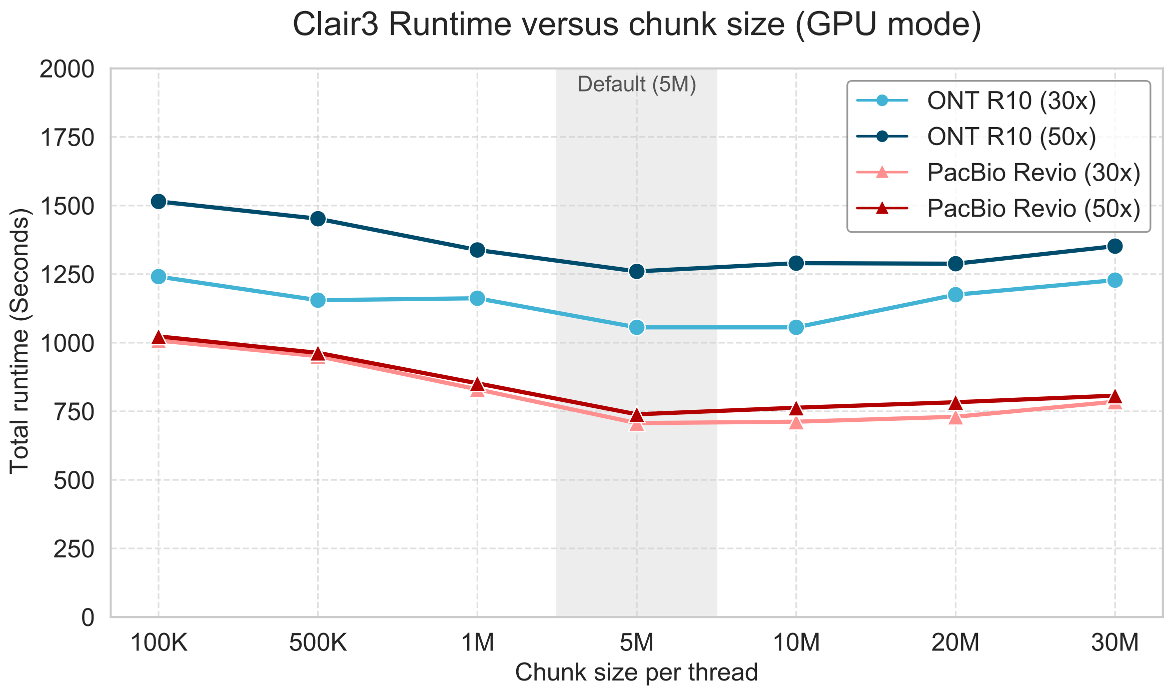

Supplementary Figure 1. Runtime comparison across different genomic chunk sizes.

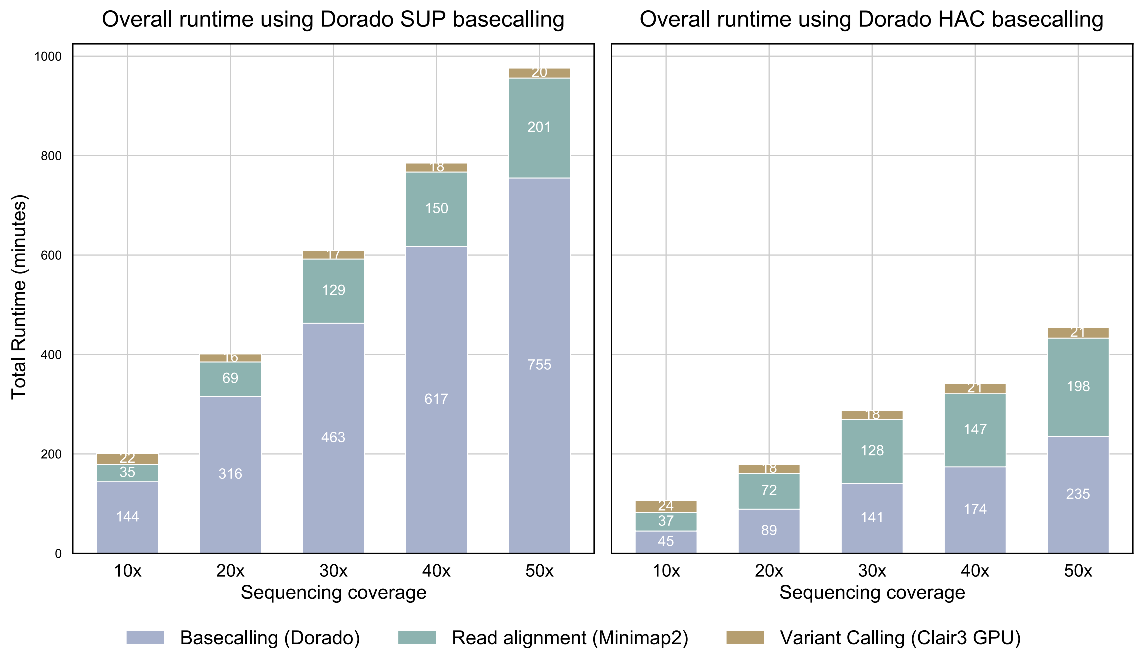

Supplementary Figure 2. Runtime overview includes the upstream basecalling, read alignment, and variant calling.

Basecalling (Dorado) was performed using 3× NVIDIA 4090 GPUs; read alignment (Minimap2) utilized 36 CPU threads; and variant calling (Clair3) was executed using 1× NVIDIA 4090 GPU.

Supplementary Tables

Supplementary Table 1. Runtime analysis of different submodules of Clair3 in ONT and PacBio platforms.

Sample	Platform	Read coverage	Pileup calling	LongPhase for variant phasing	or WhatHap for variant phasing	Full-alignment calling	Workflow control	Total runtime
HG003	ONT r10.4.1 5kHz	30x	500	72	723	396	87	1056
		50x	606	93	801	538	22	1259
	PacBio Revio	30x	359	42	653	235	70	706
		50x	357	67	826	282	33	739

The runtime is calculated in second

Supplementary Table 2. Runtime, memory, and data movement IO breakdown of different methods on HG003 datasets.

Sample	Platform	Caller	Read coverage	Runtime (minutes)	Peak memory (MB)	Average memory (MB)	Total data movement IO (GB)
HG003 WGS	ONT r10.4.1 5kHz	Clair3 v1.2.0 (GPU mode)	10x	22	45,935	10,180	137
			20x	16	56,643	6,803	175
			30x	17	57,901	10,189	222
			40x	18	62,871	6,260	263
			50x	20	63,174	8,096	307
		DeepVariant v1.10-beta (GPU mode)	10x	39	63,386	42,331	125
			20x	60	56,509	46,861	196
			30x	48	60,245	46,810	235
			40x	65	65,320	56,213	289
			50x	82	69,633	49,208	346
	PacBio Revio	Clair3 v1.2.0 (GPU mode)	10x	14	41,832	6,781	88
			20x	13	52,578	5,758	98
			30x	12	49,319	8,034	128
			40x	12	46,024	7,985	125
			50x	12	55,095	5,489	139
		DeepVariant v1.10-beta (GPU mode)	10x	34	52,412	39,789	80
			20x	40	58,852	45,697	99
			30x	40	57,350	42,140	128
			40x	48	66,953	52,545	126
			50x	53	58,773	45,738	155

Supplementary Table 3. Performance of Clair3 and DeepVariant across a range of coverages on both ONT and PacBio datasets.

Platform	Read coverage	Caller	Overall			SNP			Indel		
			Precision	Recall	F1-score	Precision	Recall	F1-score	Precision	Recall	F1-score
ONT r10.4.1 5kHz	10x	Clair3	94.78%	92.11%	93.42%	96.42%	96.05%	96.23%	81.78%	66.08%	73.09%
	10x	Clair3 pileup	90.24%	89.07%	89.65%	93.37%	93.27%	93.32%	67.90%	61.41%	64.49%
	10x	DeepVariant	95.36%	88.64%	91.88%	97.58%	92.35%	94.89%	78.74%	64.19%	70.72%
	20x	Clair3	98.53%	96.21%	97.35%	99.46%	99.20%	99.33%	91.40%	76.48%	83.28%
	20x	Clair3 pileup	96.50%	95.20%	95.85%	98.60%	98.76%	98.68%	81.17%	71.76%	76.18%
	20x	DeepVariant	96.70%	96.43%	96.56%	99.32%	99.02%	99.17%	79.83%	79.37%	79.60%
	30x	Clair3	99.03%	96.92%	97.96%	99.74%	99.42%	99.58%	93.77%	80.42%	86.58%
	30x	Clair3 pileup	97.67%	96.38%	97.02%	99.43%	99.51%	99.47%	84.97%	75.75%	80.10%
	30x	DeepVariant	97.22%	97.70%	97.46%	99.72%	99.68%	99.70%	81.78%	84.62%	83.17%
	40x	Clair3	99.20%	97.27%	98.23%	99.82%	99.47%	99.65%	94.70%	82.76%	88.33%
	40x	Clair3 pileup	97.94%	96.88%	97.41%	99.63%	99.71%	99.67%	86.08%	78.19%	81.95%
	40x	DeepVariant	97.45%	98.10%	97.77%	99.80%	99.77%	99.79%	83.13%	87.06%	85.05%
	50x	Clair3	99.28%	97.57%	98.41%	99.85%	99.55%	99.70%	95.15%	84.51%	89.52%
	50x	Clair3 pileup	98.06%	97.16%	97.61%	99.70%	99.78%	99.74%	86.66%	79.88%	83.13%
	50x	DeepVariant	97.58%	98.30%	97.94%	99.84%	99.79%	99.81%	83.92%	88.44%	86.12%
PacBio Revio	10x	Clair3	98.57%	96.66%	97.61%	99.22%	97.86%	98.54%	94.24%	88.71%	91.39%
	10x	Clair3 pileup	97.40%	95.43%	96.41%	98.93%	97.33%	98.13%	87.26%	82.89%	85.02%
	10x	DeepVariant	98.45%	95.41%	96.91%	99.26%	96.37%	97.80%	93.19%	89.07%	91.08%
	20x	Clair3	99.59%	99.28%	99.43%	99.83%	99.58%	99.70%	98.06%	97.34%	97.70%
	20x	Clair3 pileup	99.12%	98.95%	99.04%	99.77%	99.68%	99.73%	94.95%	94.16%	94.55%
	20x	DeepVariant	99.57%	99.45%	99.51%	99.84%	99.71%	99.78%	97.84%	97.74%	97.79%
	30x	Clair3	99.78%	99.69%	99.73%	99.90%	99.80%	99.85%	99.02%	98.91%	98.97%
	30x	Clair3 pileup	99.52%	99.47%	99.49%	99.88%	99.84%	99.86%	97.18%	97.02%	97.10%
	30x	DeepVariant	99.77%	99.76%	99.76%	99.90%	99.87%	99.88%	98.95%	99.00%	98.98%
	40x	Clair3	99.83%	99.78%	99.81%	99.91%	99.85%	99.88%	99.34%	99.31%	99.32%
	40x	Clair3 pileup	99.67%	99.63%	99.65%	99.91%	99.86%	99.89%	98.12%	98.11%	98.12%
	40x	DeepVariant	99.83%	99.81%	99.82%	99.91%	99.88%	99.90%	99.32%	99.36%	99.34%
	50x	Clair3	99.86%	99.83%	99.85%	99.92%	99.88%	99.90%	99.51%	99.50%	99.50%
	50x	Clair3 pileup	99.75%	99.71%	99.73%	99.93%	99.87%	99.90%	98.63%	98.67%	98.65%
	50x	DeepVariant	99.86%	99.83%	99.84%	99.92%	99.88%	99.90%	99.47%	99.50%	99.49%

Supplementary Table 4. Performance evaluation of Clair3 across multiple GIAB reference samples.

Platform	Read coverage	Sample	Caller	Overall			SNP			Indel		
				Precision	Recall	F1-score	Precision	Recall	F1-score	Precision	Recall	F1-score
ONT r10.4.1 5kHz	30x	HG001	Clair3	99.15%	97.40%	98.26%	99.78%	99.58%	99.68%	94.21%	82.19%	87.79%
		HG002	Clair3	99.00%	97.07%	98.02%	99.70%	99.65%	99.68%	93.87%	80.51%	86.68%
		HG003	Clair3	99.03%	96.92%	97.96%	99.74%	99.42%	99.58%	93.77%	80.42%	86.58%
		HG004	Clair3	99.07%	96.98%	98.01%	99.77%	99.55%	99.66%	93.86%	80.16%	86.47%
		HG005	Clair3	99.52%	98.79%	99.15%	99.82%	99.72%	99.77%	97.14%	91.46%	94.21%
		HG006	Clair3	99.38%	98.08%	98.73%	99.76%	99.69%	99.73%	96.21%	85.82%	90.71%
		HG007	Clair3	99.42%	97.86%	98.63%	99.82%	99.58%	99.70%	96.09%	84.68%	90.03%
PacBio Revio		HG002	Clair3	99.84%	99.77%	99.81%	99.92%	99.86%	99.89%	99.34%	99.21%	99.28%
		HG003	Clair3	99.78%	99.69%	99.73%	99.90%	99.80%	99.85%	99.02%	98.91%	98.97%
		HG004	Clair3	99.81%	99.73%	99.77%	99.94%	99.86%	99.90%	99.04%	98.92%	98.98%

Supplementary Table 5. Performance comparison of using LongPhase and WhatsHap for intermediate phasing.

Sample	Platform	Read coverage	Phasing method	Overall			SNP			Indel		
				Precision	Recall	F1-score	Precision	Recall	F1-score	Precision	Recall	F1-score
HG003	ONT r10.4.1 5kHz	10x	LongPhase	94.78%	92.09%	93.42%	96.41%	96.04%	96.22%	81.81%	66.07%	73.10%
		10x	WhatsHap	94.78%	92.11%	93.42%	96.42%	96.05%	96.23%	81.78%	66.08%	73.09%
		20x	LongPhase	98.52%	96.20%	97.35%	99.45%	99.20%	99.32%	91.42%	76.46%	83.28%
		20x	WhatsHap	98.53%	96.21%	97.35%	99.46%	99.20%	99.33%	91.40%	76.48%	83.28%
		30x	LongPhase	99.02%	96.90%	97.95%	99.74%	99.40%	99.57%	93.72%	80.41%	86.56%
		30x	WhatsHap	99.03%	96.92%	97.96%	99.74%	99.42%	99.58%	93.77%	80.42%	86.58%
		40x	LongPhase	99.21%	97.26%	98.23%	99.83%	99.46%	99.64%	94.69%	82.76%	88.32%
		40x	WhatsHap	99.20%	97.27%	98.23%	99.82%	99.47%	99.65%	94.70%	82.76%	88.33%
		50x	LongPhase	99.28%	97.56%	98.41%	99.86%	99.54%	99.70%	95.17%	84.48%	89.51%
		50x	WhatsHap	99.28%	97.57%	98.41%	99.85%	99.55%	99.70%	95.15%	84.51%	89.52%
	PacBio Revio	10x	LongPhase	98.58%	96.68%	97.62%	99.22%	97.89%	98.55%	94.25%	88.71%	91.40%
		10x	WhatsHap	98.57%	96.66%	97.61%	99.22%	97.86%	98.54%	94.24%	88.71%	91.39%
		20x	LongPhase	99.59%	99.29%	99.44%	99.83%	99.58%	99.70%	98.06%	97.35%	97.70%
		20x	WhatsHap	99.59%	99.28%	99.43%	99.83%	99.58%	99.70%	98.06%	97.34%	97.70%
		30x	LongPhase	99.78%	99.69%	99.73%	99.90%	99.81%	99.85%	99.02%	98.90%	98.96%
		30x	WhatsHap	99.78%	99.69%	99.73%	99.90%	99.80%	99.85%	99.02%	98.91%	98.97%
		40x	LongPhase	99.83%	99.78%	99.81%	99.91%	99.86%	99.88%	99.34%	99.31%	99.32%
		40x	WhatsHap	99.83%	99.78%	99.81%	99.91%	99.85%	99.88%	99.34%	99.31%	99.32%
		50x	LongPhase	99.86%	99.83%	99.85%	99.92%	99.88%	99.90%	99.50%	99.49%	99.50%
		50x	WhatsHap	99.86%	99.83%	99.85%	99.92%	99.88%	99.90%	99.51%	99.50%	99.50%

Supplementary Table 6. Read haplotagging comparison using different read haplotagging methods.

Platform	Haplotagging method	Read coverage	Total reads	Happlotagged reads	Happlotagged read percentage
ONT r10.4.1 5kHz	WhatsHap read haplotagging	10x	2153201	1385577	64.35%
		20x	4306362	2809059	65.23%
		30x	6461081	4226998	65.42%
		40x	8614308	5644965	65.53%
		50x	10768263	7058015	65.54%
	In-memory read haplotagging	10x	2153201	1394176	64.75%
		20x	4306362	2814720	65.36%
		30x	6461081	4234258	65.53%
		40x	8614308	5653061	65.62%
		50x	10768263	7066686	65.63%
PacBio Revio	WhatsHap read haplotagging	10x	1890290	1472882	77.92%
		20x	3781278	2995223	79.21%
		30x	5668816	4518746	79.71%
		40x	7557910	6043135	79.96%
		50x	9446519	7563783	80.07%
	In-memory read haplotagging	10x	1890290	1474215	77.99%
		20x	3781278	2999166	79.32%
		30x	5668816	4521744	79.77%
		40x	7557910	6045140	79.98%
		50x	9446519	7564809	80.08%

Command line used

BAM subsampling

Samtools(v1.10)

```
samtools view -@ ${THREADS} -s 0.${RATIO} -b -o subsampled.bam ${BAM}
samtools index -@ ${THREADS} subsampled.bam
```

Coverage calculation

Mosdepth(v0.2.9)

```
mosdepth -t ${THREADS} -n -x --quantize 0:15:150: output ${BAM}
```

Running Clair3 GPU (v 1.2)

```
bash run_clair3.sh \
-b {INPUT_DIR}/sample.bam -f ${INPUT_DIR}/ref.fa \
-m ${MODEL_PATH} \
-t ${THREAD} \
-p ${PLATFORM} \
-o ${OUTPUT_DIR} \
--use_gpu
```

Running Clair3 CPU (v 0.1 or 1.2)

```
bash run_clair3.sh \
-b {INPUT_DIR}/sample.bam -f ${INPUT_DIR}/ref.fa \
-m ${MODEL_PATH} \
-t ${THREAD} \
-p ${PLATFORM} \
-o ${OUTPUT_DIR}
```

Running DeepVariant GPU version (v1.10-beta)

```
docker run \
  -gpus 1
  -v ${INPUT_DIR}:${INPUT_DIR} \
  -v ${OUTPUT_DIR}:${OUTPUT_DIR} \
  google/deepvariant: v1.10-beta-gpu \
  /opt/deepvariant/bin/run_deepvariant \
    --model_type=${PLATFORM} \
    --ref ref.fa \
    --reads {INPUT_DIR}/sample.bam \
    --output_vcf ${OUTPUT_DIR}/output.vcf.gz \
    --num_shards ${THREAD}
```

Running DeepVariant CPU (v1.10-beta)

```
docker run \
  -v ${INPUT_DIR}:${INPUT_DIR} \
  -v ${OUTPUT_DIR}:${OUTPUT_DIR} \
  google/deepvariant:v1.10-beta \
  /opt/deepvariant/bin/run_deepvariant \
    --model_type=${PLATFORM} \
    --ref ref.fa \
    --reads {INPUT_DIR}/sample.bam \
    --output_vcf ${OUTPUT_DIR}/output.vcf.gz \
    --num_shards ${THREAD}
```

Benchmarking

hap.py (v0.3.12)

```
hap.py ${GIAB_BASELINE_VCF} output.vcf.gz \
  -o ${OUTPUT_DIR}/happy \
  -r ${REF} \
  -f ${GIAB_CONFIDENT_BED} \
  --threads ${THREADS} \
  --pass-only \
  --engine=vcfeval

# Overall insertion and deletion Precision, Recall, F1-Score
pypy3 ${REPUN} GetOverallMetrics \
  --happy_vcf_fn ${OUTPUT_DIR}/happy.vcf.gz \
  --output_fn happy.log
```

Data availability

Reference genomes

GRCh38

https://ftp.ncbi.nlm.nih.gov/genomes/all/GCA/000/001/405/GCA_000001405.15_GRCh38/seqs_for_alignment_pipelines.ucsc_ids/GCA_000001405.15_GRCh38_no_alt_analysis_set.fna.gz

GIAB truth variants

HG001 (NA12878), GRCh38, v4.2.1

https://ftp-trace.ncbi.nlm.nih.gov/giab/ftp/release/NA12878_HG001/NISTv4.2.1/GRCh38/

HG002 (NA24385), GRCh38, v4.2.1

https://ftp-trace.ncbi.nlm.nih.gov/giab/ftp/release/AshkenazimTrio/HG002_NA24385_son/NISTv4.2.1/GRCh38/

HG003 (NA24149), GRCh38, v4.2.1
https://ftp-trace.ncbi.nlm.nih.gov/giab/ftp/release/AshkenazimTrio/HG003_NA24149_father/NISTv4.2.1/GRCh38/

HG004 (NA24143), GRCh38, v4.2.1
https://ftp-trace.ncbi.nlm.nih.gov/giab/ftp/release/AshkenazimTrio/HG004_NA24143_mother/NISTv4.2.1/GRCh38/

HG005 (NA24631), GRCh38, v4.2.1
https://ftp.ncbi.nlm.nih.gov/ReferenceSamples/giab/release/ChineseTrio/HG005_NA24631_son/NISTv4.2.1/GRCh38/

HG006 (NA24694), GRCh38, v4.2.1
https://ftp.ncbi.nlm.nih.gov/ReferenceSamples/giab/release/ChineseTrio/HG006_NA24694_father/NISTv4.2.1/GRCh38/

HG007 (NA24695), GRCh38, v4.2.1
https://ftp.ncbi.nlm.nih.gov/ReferenceSamples/giab/release/ChineseTrio/HG007_NA24695_mother/NISTv4.2.1/GRCh38/

ONT sequencing Data
ONT EPI2ME Labs HG001-HG007 R10.4.1 5kHz, GRCh38
https://epi2me.nanoporetech.com/giab-2025.01

PacBio Revio sequencing Data
PacBio Revio HG002-HG004, GRCh38
https://downloads.pacbcloud.com/public/revio/2022Q4/
